# Genome‐wide characterization of 5‐hydoxymethylcytosine in melanoma reveals major differences with nevus

**DOI:** 10.1002/gcc.22837

**Published:** 2020-02-13

**Authors:** Catarina Salgado, Jan Oosting, Bart Janssen, Rajiv Kumar, Nelleke Gruis, Remco van Doorn

**Affiliations:** ^1^ Department of Dermatology Leiden University Medical Center Leiden The Netherlands; ^2^ Department of Pathology Leiden University Medical Center Leiden The Netherlands; ^3^ GenomeScan B.V. Leiden The Netherlands; ^4^ Division of Molecular Genetic Epidemiology German Cancer Research Center Heidelberg Germany

**Keywords:** 5‐hydroxymethylcytosine, DNA hydroxymethylation, DNA methylation, melanoma, *PTEN* gene

## Abstract

Melanoma demonstrates altered patterns of DNA methylation that are associated with genetic instability and transcriptional repression of numerous genes. Active DNA demethylation is mediated by TET enzymes that catalyze conversion of 5‐methylcytosine (mC) to 5‐hydroxymethylcytosine (hmC). Loss of hmC occurs in melanoma and correlates with disease progression. Here we analyzed the genomic distribution of hmC along with mC in nevus and melanoma using oxidative bisulfite chemistry combined with high‐density arrays. HmC was enriched relative to mC at enhancers, 5′UTR regions and CpG shores in nevus and melanoma samples, pointing to specific TET enzyme activity. The proportion of interrogated CpG sites with high hmC levels was lower in melanoma (0.54%) than in nevus (2.0%). Depletion of hmC in melanoma was evident across all chromosomes and intragenic regions, being more pronounced in metastatic than in non‐metastatic tumors. The patterns of hmC distribution in melanoma samples differed significantly from those in nevus samples, exceeding differences in mC patterns. We identified specific CpG sites and regions with significantly lower hmC levels in melanoma than in nevus that might serve as diagnostic markers. Differentially hydroxymethylated regions localized to cancer‐related genes, including the *PTEN* gene promoter, suggesting that deregulated DNA hydroxymethylation may contribute to melanoma pathogenesis.

## 1 INTRODUCTION

Cutaneous melanoma is a malignant tumor derived from melanocytes residing in the skin. Clinically melanoma needs to be distinguished from melanocytic nevus, a benign lesion composed of melanocytes in a stable growth arrest.^1^ Integrative genomic and transcriptomic analysis has identified common mutations and recurrent signaling perturbations yielding insight into melanoma biology.^2^ In addition to accumulated genetic alterations, epigenetic mechanisms drive the development and evolution of melanoma.^3^, ^4^ DNA methylation, histone modifications and chromatin remodeling complexes regulate chromatin accessibility to transcription factors, thereby controlling gene expression programs. DNA methylation at CpG dinucleotides is mediated by DNA methyltransferases and additionally governed by DNA demethylation. Passive DNA demethylation can occur through insufficient methyltransferase activity during replication. Active demethylation involves the oxidation of 5‐methylcytosine (mC) to 5‐hydroxymethylcytosine (hmC) performed by the Ten Eleven Translocase (TET) family of dioxygenase enzymes.^5^ In mammalian cells approximately 4% of all cytosines are methylated, and depending on cell type 0.1%‐0.7% of cytosine bases are hydroxymethylated.^6^ Epigenetic deregulation is a universal characteristic of malignant tumors implicated in tumorigenesis. Cancer genomes are characterized by widespread loss of DNA methylation that contribute to genomic instability, and gain of DNA methylation at promoter CpG islands is associated with transcriptional repression^7^ In melanoma, selected tumor suppressor genes with a critical role in malignant transformation and metastatic behavior, including *CDKN2A*, *PTEN*, and *CDH11*, show frequent promoter hypermethylation and associated transcriptional silencing.^8^ In addition, variation of methylation density at enhancer regions contributes to melanoma cell plasticity and correlates with patient survival.^9^

Different tumor types demonstrate loss of DNA hydroxymethylation and in certain instances this epigenetic event can be attributed to mutations in *TET* or *IDH* genes. Although the functional relevance of hmC loss remains to be resolved, studies in melanoma suggest its involvement in tumor progression.^10^ Accordingly, low hmC levels were associated with worse survival from melanoma. Thus, in melanoma and other tumor types hmC loss might have diagnostic as well as prognostic significance. Hydroxymethylation mapping of melanoma samples using hydroxymethylated DNA immunoprecipitation showed hmC clusters in gene‐rich regions and loss at specific loci.^10^ In glioblastoma hmC depletion was shown to be most pronounced at enhancer regions.^11^ To understand the functional consequences of aberrant hydroxymethylation and to apply it in the diagnosis and prognosis of melanoma, it is essential to obtain precise maps of the distribution of this epigenetic mark. Here we characterized the genomic distribution of hmC and mC in nevus and melanoma using oxidative bisulfite chemistry combined with arrays that simultaneously interrogate hmC and mC at 850000 CpG sites. This methodology is not affected by bias associated with antibody‐based DNA capture methods and provides robust estimates of hmC and mC.^12^ We sought to identify differentially hydroxymethylated CpG sites and regions by comparing nevus and melanoma hmC patterns. In addition, we compared the hmC patterns between primary melanoma samples that differ with respect to metastatic behavior. The genomic landscapes of hmC show depletion of hydroxymethylation in melanoma across various intragenic and intergenic regions compared to nevus. The hydroxymethylation patterns show more differences between nevus and melanoma than the methylation patterns, which has potential implications for biomarker discovery.

## 2 MATERIALS AND METHODS

### 2.1 Patient samples

Fresh‐frozen biopsy samples were obtained from patients diagnosed with common nevus (n = 8), non‐metastatic primary melanoma (n = 8), and metastatic primary melanoma (n = 8) (Table S1). Only tissue samples containing at least 50% nevus or melanoma cells were included. Genomic DNA from all samples was extracted using the Genomic‐tip kit (Qiagen, Hilden, Germany). The study was approved by the Leiden University Medical Center institutional ethical committee (05‐036) and was conducted according to the Declaration of Helsinki Principles.

### 2.2 Bisulfite and oxidative bisulfite conversion and hybridization

Genomic DNA (1 μg) was subjected to BS and OxBS conversion using the TrueMethyl 96 Kit (CEGX, Cambridge, UK) and applied to the Infinium MethylationEPIC BeadChip Kit (Illumina, San Diego, CA, USA) at GenomeScan (Leiden, The Netherlands). The BeadChip images were scanned on the iScan system and the data quality was assessed using the R script MethylAid.^13^

### 2.3 850 K beadchip data analysis

Data were processed using the ChAMP package,^14^, ^15^ normalized using the default BMIQ algorithm and analyzed as described previously with genome build GRCh37/hg19.^12^ The ratio of the signal for the cytosine sequence to the combined intensity is the *β* value, reflecting the methylation level on a scale from 0 (unmethylated) to 1 (fully methylated). To obtain the hydroxymethylation fraction oxBS *β* values are subtracted from BS beta values, generating Δ*β* values.^12^, ^16^ To define CpGs with high hmC we established a cutoff based on the average of absolute Δ*β* value for all probes (0.008 + 3 SDs, 0.166). To compare groups (nevus vs melanoma; nonmetastatic vs metastatic melanoma) a statistical test using the Limma R package^17^ was used with multiple testing corrections applying a stringent *P* value <.005.^18^ The Bump Hunting Algorithm was used to identify differentially hydroxymethylated regions with closely positioned probes.^19^ The rate of hmC, the average of Δ*β* values for a specific group of CpGs, was calculated according to intragenic location, to CpG‐context regions and at enhancer regions (melanocytic cell‐specific and general) retrieved from FANTOM5 project (http://FANTOM5.gsc.riken.jp/5/).^20^

### 2.4 Validation of candidate loci

Validation of hydroxymethylation at the *PTEN* promoter was performed in an independent sample group (four nevi and four melanoma metastases). Genomic DNA (1 μg) was subjected to BS and OxBS conversion using TrueMethyl oxBS Module (NuGEN Technologies, Redwood City, CA, USA). DNA was amplified using the PCR_X_ Enhancer System (Thermo Fisher Scientific, Waltham, MA, USA) and subjected to capillary sequenced (primers: GGGGTTGTAAATAGATTTGATAGG and AAAAATATCTCCTACTACAACCCAAAA) and deep paired‐end sequencing (tailed primers: GATGTGTATAAGAGACAGGGGGTTGTAAATAGATTTGATAGG and CGTGTGCTCTTCCGATCTAAAAATATCTCCTACTACAACCCAAAA) using a MiSeq system (Illumina).

## 3 RESULTS

### 3.1 Obtaining genome‐wide 5‐hydroxymethylcytosine patterns

Twenty‐four DNA samples were analyzed, including eight aggressive primary melanomas with metastatic behavior (M+), eight primary melanomas with no metastatic behavior (M−) during long‐term follow‐up, and eight benign nevi (N) (Table 1, Table S1). To detect methylcytosine (mC) and hydroxymethylcytosine (hmC), different states of the CpG sites, we applied oxidative bisulfite (oxBS) chemistry, calculating hmC levels based on differences between bisulfite (BS) and oxBS‐treated samples, using arrays as described previously.^11^, ^12^, ^21^ Bisulfite (BS) converts unmethylated cytosines to uracil, while methylated and hydroxymethylated cytosines are protected. The prior oxidative step in oxBS conversion allows the distinction between methylated and hydroxymethylated cytosines. Only hydroxymethylated but not methylated cytosines are oxidated into formylcytosines (5fC), which are converted to uracil. Arrays that interrogate over 850 000 CpG sites representing 99% of the RefSeq genes, encompassing more than 90% of interrogated sites of 450 K arrays plus 333,265 CpGs located at enhancer regions were used.^22^ After quality control and exclusion of X‐chromosomal CpGs 743,016 CpGs were analyzed. As a measure of DNA methylation, the fluorescence ratio (*β* value, ranging from 0 to 1) for each CpG of the bisulfite‐treated DNA sample was used. Subtraction of the normalized *β* value of the oxBS‐treated sample from that of the BS‐treated replicate analyzed in parallel (Δ*β* value) was used as a measure of hydroxymethylation (Figure S1). The average Δ*β* value for CpGs at different genomic locations (hmC rate) was calculated. In addition, we considered as CpGs with high hmC levels those having a Δ*β* value exceeding the average plus three SDs (Δ*β* > 0.166).

**Table 1:** Clinical characteristics of nevus and melanoma samples subjected to genome‐wide DNA (hydroxy)methylation analysis

	Melanocytic Nevi n = 8	Non‐metastatic primary melanomas n = 8	Metastatic primary melanomas n = 8
Gender			
Female	6	4	4
Male	2	4	4
Age at diagnosis in years, median (range)	42 (29‐57)	39 (34‐68)	63 (45‐79)
Location			
Head/neck	4	1	3
Trunk	2	4	2
Extremities	2	3	3
Breslow depth in mm, median (range)		1.0 (0.73‐4)	9.7 (1.9‐17)

**Figure 1:**
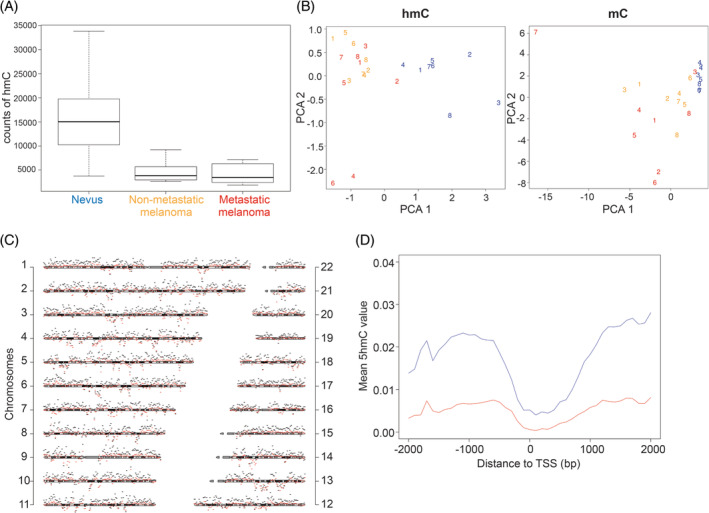
Genome‐wide distribution of DNA hydroxymethylation in nevus, nonmetastatic and metastatic melanoma. (A) Boxplot showing the counts of CpGs with high hmC (Δ*β* > 0.166) for each group. (B) Principal component analysis of hmC and mC for 1% of probes with highest variation across samples. Numbers refer to individual samples. Blue—nevi; yellow—non‐metastatic melanomas; red—metastatic melanomas. (C) Chromosomal distribution of hmC in nevi (black) and melanomas (red). The scheme of each chromosome represents the measurement baseline (null hmC level), the vertical distance between chromosomes is 10%, bin size is 1 Mb. (D) Mean of hmC level over 4 Kb around the transcription start sites for nevi (blue) and melanomas (red)

First, we compared the number of hydroxymethylated CpGs in the nevus, non‐metastatic and metastatic melanoma sample groups. The number of CpGs with high hmC levels was significantly higher in nevus (2.0% of interrogated CpGs) than in melanoma (0.54%) samples as well as the average Δ*β* value for the sample groups (0.017 vs 0.004), consistent with earlier reports of hmC loss in melanoma (Figure 1A)^10^ Comparative analysis of melanoma and nevus samples revealed 21,767 CpGs with significantly lower hydroxymethylation in melanoma than in nevus samples, whereas 397 CpGs showed higher levels of hmC in melanoma (FDR <0.005). However, the variation of hmC levels of these CpGs within sample groups was high (Figure S2). In spite of heterogeneity certain CpG sites showed consistent hmC loss in melanoma. The 50 most differentially hydroxymethylated CpGs are presented in a heatmap in Figure S3. When comparing metastatic and nonmetastatic primary melanoma samples there were no interrogated CpGs with statistically significant different hmC level.

To capture the distribution of hmC, principal component analysis revealed that the hmC patterns of melanoma samples were distinct from those of nevus samples (Figure 1B). The differences between the sample groups were more pronounced for hmC than for mC patterns. The hmC patterns of metastatic and nonmetastatic melanoma samples were not distinct in this analysis. The hmC levels at different chromosomal regions were almost uniformly higher in nevus than in melanoma samples, with no evident clustering of aberrant hmC at specific chromosomal regions (Figure 1C).

### 3.2 Depletion of hmC in different genomic regions

**Figure 2:**
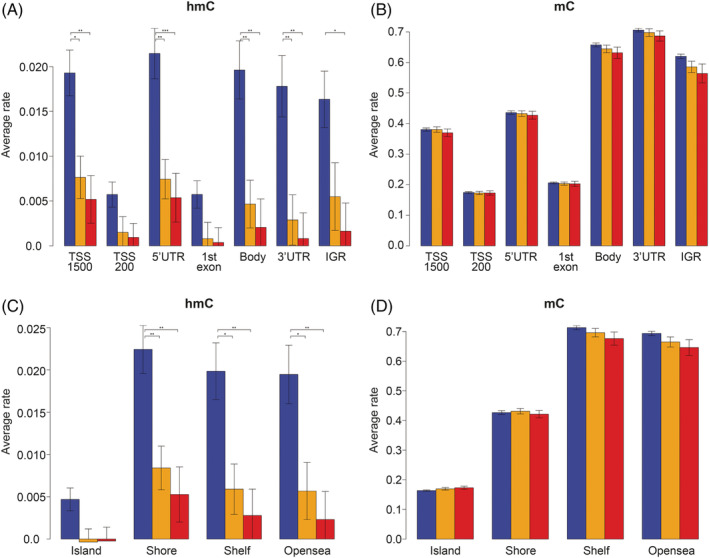
Average rate of hmC and mC at intragenic locations and CpG‐context regions. (A) hmC levels in the N, M−, and M+ sample groups at intragenic regions presented as average Δ*β* values. (B) mC level presented as average *β* values. Untranslated regions (3′UTR and 5′UTR), proximal promoter (TSS‐200 bp and 1^st^exon), distal promoter (TSS‐1500 bp), gene body, and intergenic region (IGR). (C) hmC levels in the N, M−, and M+ sample groups at CpG‐context regions presented as average Δ*β* values. (D) mC level presented as average *β* values. CpG island, shore (<2 Kb flanking CpG Islands), shelves (<2 Kb flanking outwards from CpG shore) and open sea (>4 Kb from CpG island). Blue—nevi; yellow—non‐metastatic melanomas (M−); red—metastatic melanomas (M+). The error bars represent standard errors among samples

Since methylation of promoter, intragenic, and intergenic regions has distinct associations with gene transcription, we determined the location of hmC and mC within these regions. First, we assessed the average hmC rate across 4 Kb at promoter regions around the canonical transcription start site of all genes and observed slightly lower hmC levels in melanoma throughout the entire region compared to benign nevus (Figure 1D). TET proteins generate hmC as an intermediate from mC in active DNA demethylation; hmC levels tend to follow mC levels therefore. Accordingly, both mC and hmC levels were considerably lower at CpGs in the proximal promoter and first exon. However, the distal promoter (200‐1500 bp upstream of transcription start site) and 5′UTR regions are exceptions that show high hmC in spite of moderate mC levels in all sample groups (Figure 2A,B). Whereas the mC levels were only marginally lower in melanoma than in nevus, we observed a striking loss of hmC not only in promoters but across all gene regions. The levels of hmC were also significantly lower in the metastatic than in the nonmetastatic melanomas in most gene regions.

Higher variation of hmC at enhancer regions in tumor has been reported in glioblastoma.^11^ Therefore, we analyzed the average rate of hmC at melanocyte‐specific and at general enhancer regions retrieved from the FANTOM5 project.^20^ We found higher hmC levels in enhancer compared to non‐enhancer regions among the different sample groups (Figure S4). The depletion of hmC at enhancer regions in melanoma compared with nevus was proportional to that at non‐enhancer regions.

CpG islands, particularly located at promoter regions, are mostly protected from methylation. The regions adjacent to CpG islands, termed shores and shelves have also been found to demonstrate specific methylation patterns associated with transcriptional states.^23^, ^24^ Subsequently we calculated the hmC and mC levels of cytosines located in these regions and found that the mC levels were lower in CpG islands and shores than in shelves and open sea (Figure 2C,D). Again, the loss of hmC in melanoma compared to nevus was much larger than the difference in mC across the CpG islands, shores, shelves and open sea. Whereas generally the hmC levels follow the mC levels, the CpG shores are another exception demonstrating high hmC in spite of moderate mC levels, especially in nevus samples.

Taken together, in nevus and melanoma hmC levels differ markedly across genomic regions and not following mC levels, which points to specific enzymatic activity in shaping hmC patterns. The hmC levels are substantially lower in melanoma than in nevus across all intragenic regions. This is in line with dilution through replication and insufficient active TET‐mediated hydroxymethylation. Differences of hmC levels and distribution are much more pronounced than of mC levels.

### 3.3 Differentially hydroxymethylated regions in melanoma

**Figure 3:**
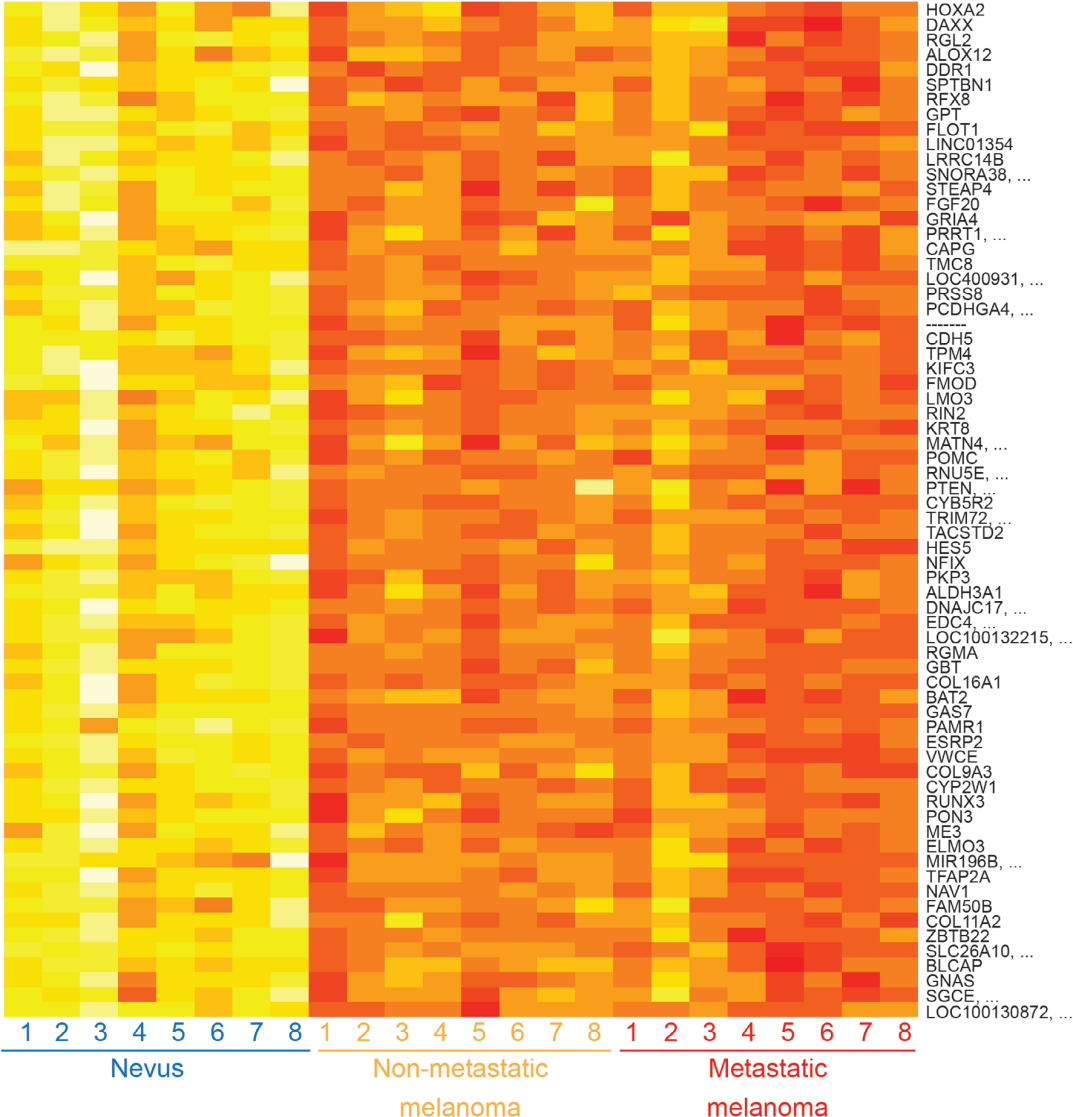
Heatmap depicting hmC levels for 68 significantly differentially hydroxymethylated regions in nevus and melanoma samples. Each row represents a DhMR with the associated gene and each column represents a different sample. Average hmC level, measured as Δ*β* value, is indicated by variable color (low hmC—red, high hmC—yellow)

Although the modification of a single CpG site may impact on gene expression, regions containing multiple CpG sites in promoters and enhancers commonly work as units of transcriptional regulation. Therefore, we sought to identify and examine regions with differential hydroxymethylation (DhMRs). When comparing melanoma and nevus samples, 68 regions were statistically significant differentially hydroxymethylated (*P* < .005). In all 68 DhMR hmC levels were lower in melanoma compared to nevus (Figure 3, Table S2). No significantly differentially hydroxymethylated regions were identified when comparing metastatic and non‐metastatic melanoma samples. Five of these regions are located within established cancer‐related genes (http://cancer.sanger.ac.uk/census, accessed October 2019), namely in the *GNAS*, *GAS7*, *PTEN*, *TPM4*, and *DAXX* (Table S2). Notably, for the *PTEN* and *TPM4* tumor suppressor genes the DhMR is located in the promoter region.

### 3.4 *PTEN* promoter hydroxymethylation in nevus and melanoma

**Figure 4:**
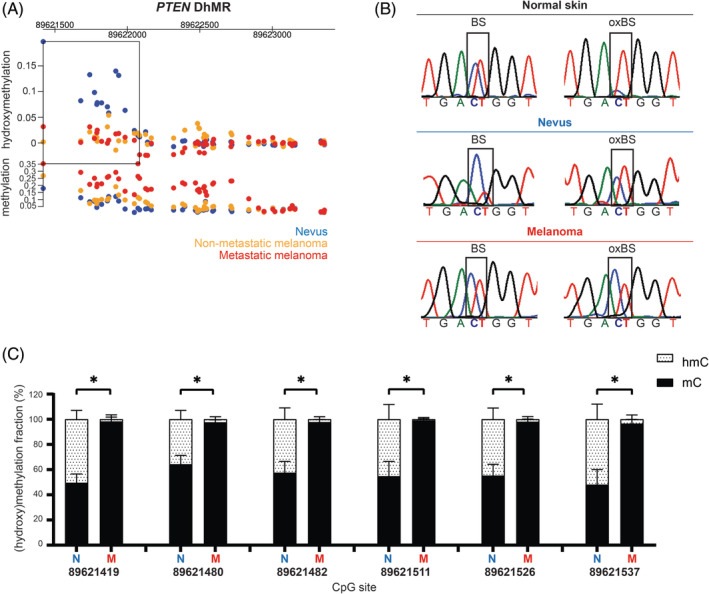
Differentially hydroxymethylated region in the *PTEN* promoter region (A) DhMR (rectangle; chr10:89621419‐89622084) located within the promoter region of *PTEN* gene with hydroxymethylation and methylation levels for the three sample groups. (B) Validation of a selected CpG site from *PTEN* DhMR by capillary sequencing upon BS and oxBS conversion. After oxBS a higher T peak appears in normal skin and nevus samples, while in melanoma sample there is a higher C peak. (C) BS/OxBS deep sequencing of six CpG sites at the *PTEN* DhMR (chr10:89621419‐89621537) in four independent nevi and four melanomas (mean ± SD, *P < .05, two‐tailed Mann‐Whitney *U* test). Blue—nevi; yellow—nonmetastatic melanomas; red—metastatic melanomas

*PTEN* is an established tumor suppressor gene, inactivated in melanoma and other tumor types through genetic and epigenetic mechanisms. Therefore, we further analyzed hydroxymethylation at this locus in nevus and melanoma. In our study, a region in the *PTEN* promoter (chr10:89621419‐89622084) was found to show hydroxymethylation in all nevus samples, but higher methylation levels in the melanoma samples (Figure 4A). Methylation of this specific region in the *PTEN* promoter, located from −1400 to −800 bp upstream of the transcription start site, has been reported as being associated with transcriptional repression of *PTEN* in various malignancies and worse survival in melanoma patients (Figure S5).^25^, ^26^, ^27^ Capillary sequencing of the region following BS and oxBS conversion of DNA from nevus and melanoma samples subjected to hmC profiling, along with a normal skin sample, confirmed the presence of hydroxymethylation in nevus and normal skin samples (higher T peak upon oxBS) and methylation in a melanoma sample (maintenance of higher C peak after Bs and oxBS) (Figure 4B). Next, we analyzed this DhMR in the *PTEN* promoter using an independent quantitative BS/oxBS deep sequencing method in an independent set of four nevi and four metastatic melanoma samples. The six CpGs analyzed using BS/oxBS NGS (chr10:89621419‐89621537) confirmed the hydroxymethylation profile in nevi and a predominant methylation status in melanomas (Figure 4C).

## 4 DISCUSSION

Loss of hmC is an established feature of melanoma and other tumor types, with potential diagnostic and prognostic significance.^10^, ^28^ Here we provide a genome‐wide landscape of hmC and mC in nevus and melanoma by applying robust oxidative bisulfite chemistry combined with high‐density arrays. Unsupervised analysis revealed significant differences in the global hmC patterns of melanoma and nevus samples, exceeding those of mC patterns. Numerous published studies have aimed to identify diagnostic and prognostic DNA methylation markers for melanoma.^29^, ^30^, ^31^ Our study shows that analysis of hmC levels and distribution can equally be used to aid in distinguishing melanoma from benign melanocytic lesions. Accordingly, determination of hmC levels using immunohistochemistry in the diagnosis of melanoma has been proposed.^32^ We identified thousands of single differentially hydroxymethylated CpG sites and 68 regions that might be used as specific diagnostic markers for melanoma. Although the levels of hmC were uniformly lower in metastatic than in non‐metastatic melanoma, the patterns of distribution were not significantly different.

We observed a striking loss of hmC in melanoma relative to nevus, consistent with findings in other tumor types, across all autosomes, intragenic and intergenic regions, within and outside of CpG islands.^11^, ^33^ This phenomenon may be explained by passive dilution of the hmC mark due to DNA replication in proliferating melanoma cells and by insufficient active demethylation. Downregulation of IDH and TET family enzymes in melanoma has been shown previously, involving deregulation of active TET‐mediated DNA demethylation in shaping the melanoma epigenome.^10^ Within the pattern of global hmC depletion, specific CpG sites and regions could be identified with significantly lower hydroxymethylation in melanoma than in nevus, pointing to epigenetic deregulation at specific loci. In nevus and melanoma, the hydroxymethylation levels were particularly low at the proximal promoter (TSS200) and first exon, corresponding with lower levels of methylation at promoter CpG islands. However, at CpG shores we observed high levels of hydroxymethylation disproportionate to the methylation levels at these sites in nevus and melanoma. Enrichment of hmC at CpG shores, regions that regulate gene expression, has been reported in non‐small cell lung cancer and liver cancer previously.^24^

In melanoma and other tumor types, the methylation landscape demonstrates marked alterations at enhancer regions, which can impact on gene expression programmes and tumor aggressiveness.^9^ Oxidation of mC into hmC is associated with enhancer activation.^34^ Hydroxymethylation at these critical regulatory regions in tumors could induce functional demethylation and activation. In this study, we observed enrichment of hmC at enhancer regions in nevus and melanoma, as was reported for glioblastoma, but no excess depletion of hmC at enhancers in melanoma.^11^

The hmC mark is associated with an open chromatin configuration, affecting gene expression regulation.^34^ Active demethylation can protect promoter and enhancer regions from methylation‐associated silencing. Loss of hmC might therefore contribute to malignant progression. Among the 68 DhMRs, five localized to the cancer‐related genes *PTEN, DAXX*, *GAS7*, *GNAS*, and *TPM4*. *PTEN* is an essential tumor suppressor gene in melanoma. Here, we demonstrate the presence of hydroxymethylation in the promoter region of the *PTEN* gene (chr10:89621419‐89622084) in nevi and its absence in melanomas. It has been reported that *PTEN* expression is uniformly high in nevus and markedly lower in melanoma samples.^10^, ^35^, ^36^ In melanomas, *PTEN* is functionally inactivated through genetic and epigenetic mechanisms, including promoter hypermethylation.^25^, ^37^ Loss of *PTEN* expression in murine nevi accelerates melanoma formation by allowing escape from oncogene‐induced senescence.^38^ It is tempting to speculate that hmC depletion at the *PTEN* regulatory region in melanoma has functional significance by affecting expression of this tumor suppressor gene. Accordingly, it was recently found that ablation of the *TET2* gene, resulting in genomic hmC loss, drives malignant transformation and melanoma progression.^39^ In the genetically engineered mouse models studied deregulated expression of *CDKN2A* was observed. Even partial *PTEN* loss due to epigenetic mechanisms has biological relevance in melanoma.^36^ Of note, the CpG sites showing hypermethylation in the study by Giles et al^36^ are located within the DhMR we identified. The potential role of depletion of hmC at the *PTEN* promoter as an epigenetic mechanism driving melanoma progression requires further investigation.

In conclusion, we have resolved the genome‐wide hmC and mC distribution in melanoma and nevus, of potential relevance for biomarker discovery and understanding of epigenetic deregulation in melanoma. We identified specific CpG sites and regions with significantly lower hydroxymethylation in melanoma than in nevus. Our results merit further investigation into the functional relevance of hydroxymethylation at the *PTEN* promoter in nevus and depletion at this locus in melanoma. Methods used in previous studies to analyze DNA methylation that rely on bisulfite conversion may have overestimated methylation, since part of the observed protection from conversion to uracil is caused by hydroxymethylation. However, we can assume that this potential error on melanoma is minor. Following on this genome‐wide analysis of hmC, the value of the identified differentially hydroxymethylated CpG sites and regions should be tested in a large cohort of dysplastic melanocytic nevi and melanomas.

## CONFLICT OF INTEREST

The authors declare no conflicts of interest.

## Supporting information


**Figure S1** Cumulative distribution of hmC and mC across all CpG sites analyzed. Red line for hmC; black line for mC. All CpG sites show a hmC value between −0.2 and 0.3. The mC distribution is bimodal since there are nonmethylated CpGs (0‐0.2) or fully methylated CpGs (0.8‐1).
**Figure S2**. Venn diagram of the GC‐probes for which at least one sample within a group showed a Δ*β* value exceeding the average plus three SDs (Δ*β* > 0.166).
**Figure S3**. Heatmap. The top 50 CpG sites statistically significant between nevi and melanomas in order of hmC value.
**Figure S4**. Averaged rate of hmC at enhancer regions retrieved from FANTOM5 project (http://FANTOM5.gsc.riken.jp/5/). Blue—nevi; yellow—nonmetastatic melanoma; red—metastatic melanoma. Comparison of hmC rate at melanocyte‐specific enhancer regions (2) (2593 probes were found in 2136 enhancers) and at general enhancer regions (1) with hmC rate at nonenhancer regions (0).
**Figure S5**. Schematic representation of the DhMR in the PTEN promoter region (chr10:89621419‐89622084). Hypermethylation of the regions (1) (Mirmohammadsadegh et al^25^) and (2) (Lahtz et al^26^) have been previously associated with transcriptional repression of the *PTEN* gene in melanoma. Hypermethylation of region (3) (Roh et al,^27^ same as region (1)) was associated with worse survival in melanoma patients.

**Table S1** Tumor samples characteristics.

**Table S2** Sixty‐eight differentially hydroxymethylated regions (DhMRs).

## Data Availability

The data that support the findings of this study are available from the corresponding author upon reasonable request.
