# Supplementary material for: Genome‐wide characterization of 5‐hydoxymethylcytosine in melanoma reveals major differences with nevus
Source: Genes Chromosomes Cancer. 2020 Feb 13;59(6):366–74. doi: 10.1002/gcc.22837 (PMC7318264; doi:10.1002/gcc.22837)
Supplement: Supplementary file 1 — Figure S1 Cumulative distribution of hmC and mC across all CpG sites analyzed. Red line for hmC; black line for mC. All CpG sites show a hmC value between −0.2 and 0.3. The mC distribution is bimodal since there are nonmethylated CpGs (0‐0.2) or fully methylated CpGs (0.8‐1). Figure S2. Venn diagram of the GC‐probes for which at least one sample within a group showed a Δβ value exceeding the average plus three SDs (Δβ > 0.166). Figure S3. Heatmap. The top 50 CpG sites statistically significant between nevi and melanomas in order of hmC value. Figure S4. Averaged rate of hmC at enhancer regions retrieved from FANTOM5 project (http://FANTOM5.gsc.riken.jp/5/). Blue—nevi; yellow—nonmetastatic melanoma; red—metastatic melanoma. Comparison of hmC rate at melanocyte‐specific enhancer regions (2) (2593 probes were found in 2136 enhancers) and at general enhancer regions (1) with hmC rate at nonenhancer regions (0). Figure S5. Schematic representation of the DhMR in the PTEN promoter region (chr10:89621419‐89622084). Hypermethylation of the regions (1) (Mirmohammadsadegh et al25) and (2) (Lahtz et al26) have been previously associated with transcriptional repression of the PTEN gene in melanoma. Hypermethylation of region (3) (Roh et al,27 same as region (1)) was associated with worse survival in melanoma patients. [file GCC-59-366-s001.docx]

**Supplemental figures**


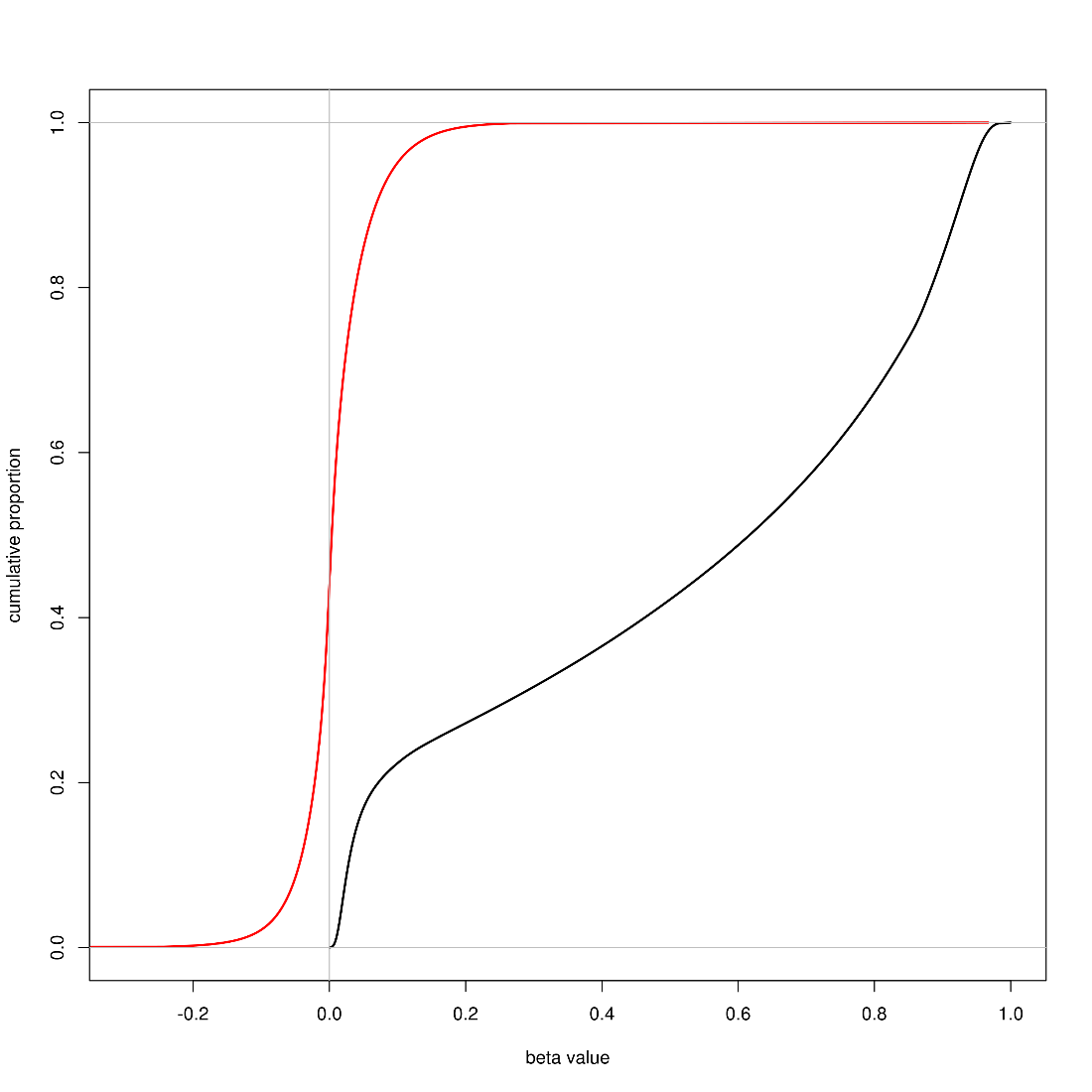


mc

hmc

**Figure S1.** **Cumulative distribution of hmC and mC across all CpG sites analysed.** Red line for hmC; black line for mC. All CpG sites show a hmC value between -0.2 and 0.3. The mC distribution is bimodal since there are non-methylated CpGs (0-0.2) or fully methylated CpGs (0.8-1).

**
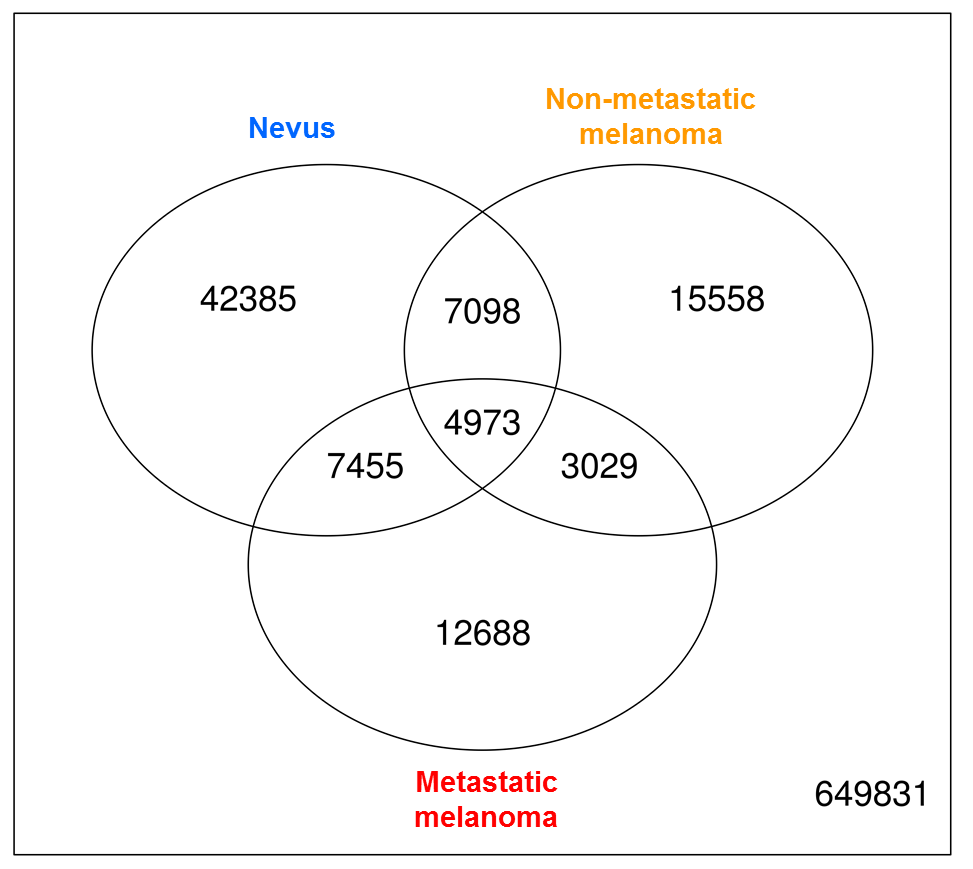
**

**Figure S2**. Venn diagram of the GC-probes for which at least 1 sample within a group showed a Δβ value exceeding the average plus 3 standard deviations (Δβ>0.166).


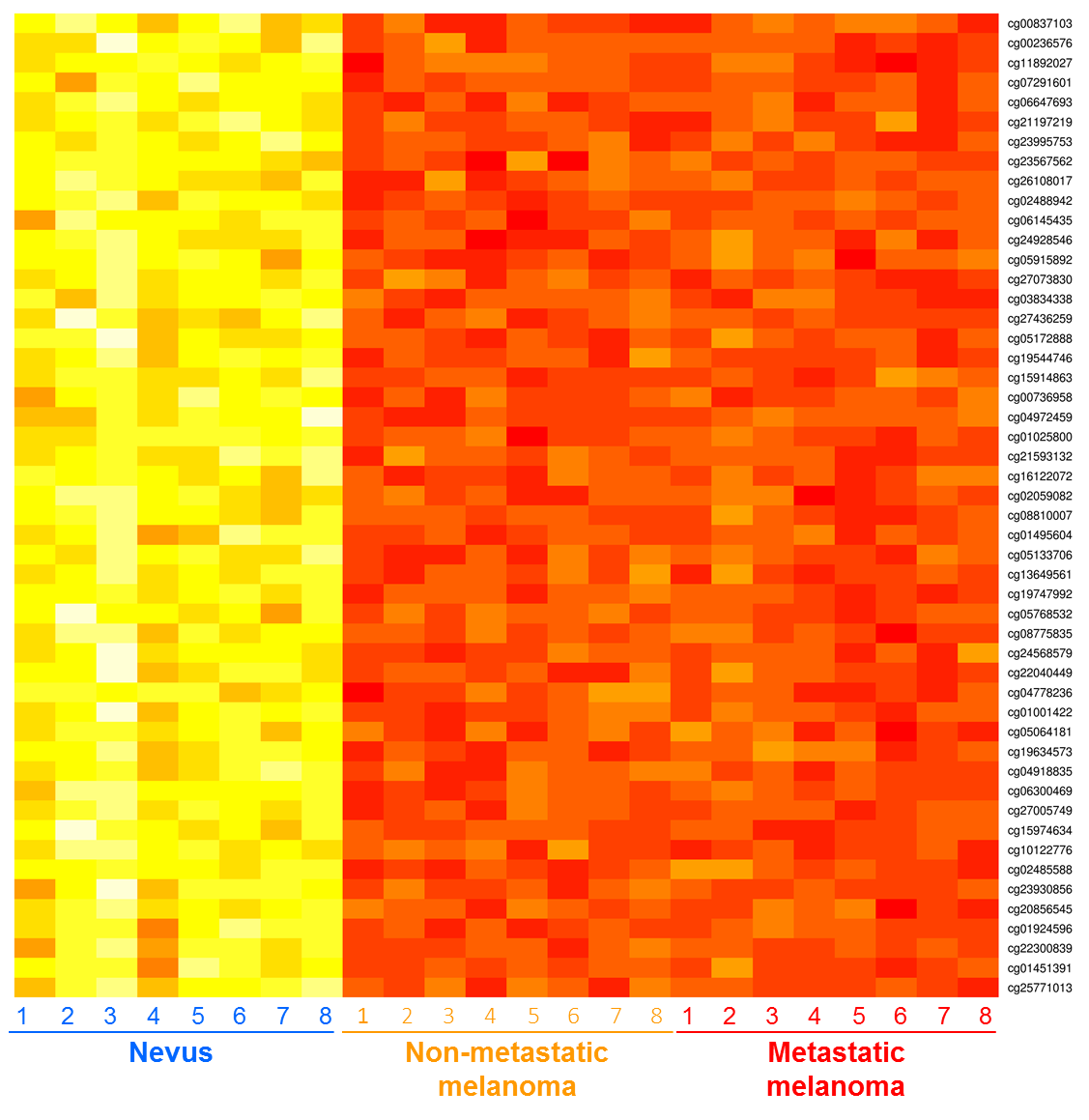


**Figure S3**. **Heatmap.** The top50 CpG sites statistically significant between nevi and melanomas in order of hmC value.

**
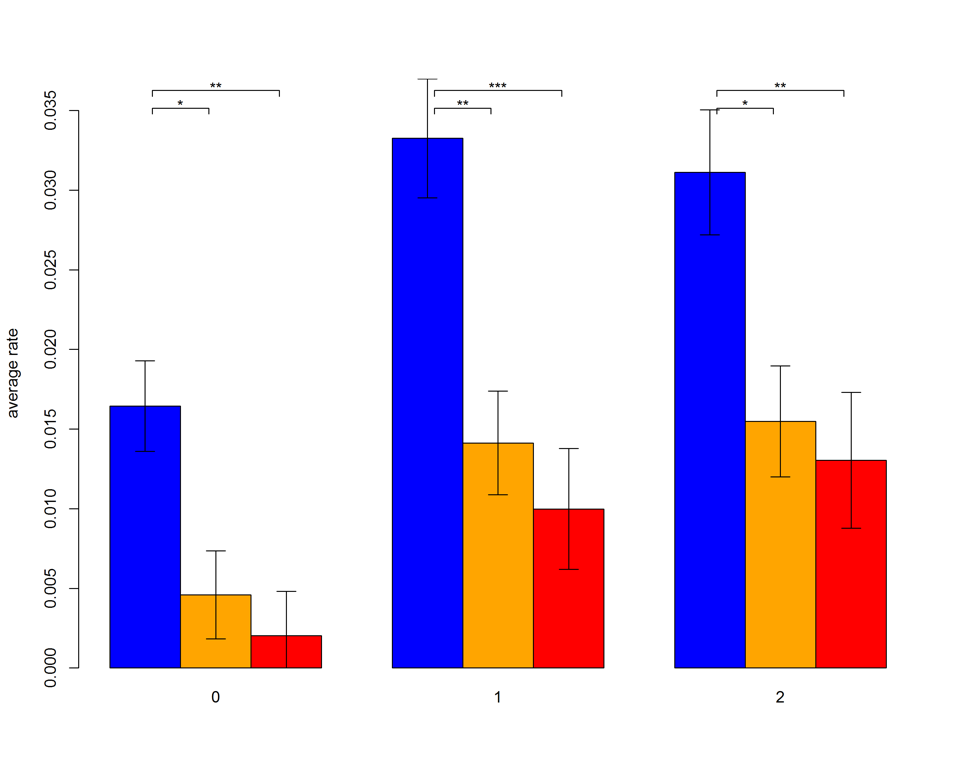
**

**Figure S4**. **Averaged rate of hmC at enhancer regions retrieved from FANTOM5 project** ([http://FANTOM5.gsc.riken.jp/5/](http://fantom.gsc.riken.jp/5/)). Blue – nevi; yellow – non-metastatic melanoma; red – metastatic melanoma. Comparison of hmC rate at melanocyte-specific enhancer regions (2) (2593 probes were found in 2136 enhancers) and at general enhancer regions (1) with hmC rate at non-enhancer regions (0).

**
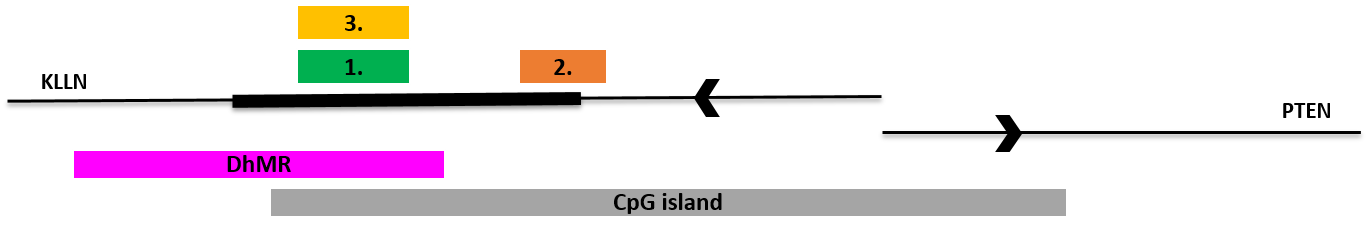
**

**Figure S5**. **Schematic representation of the DhMR in the PTEN promoter region (chr10:89621419-89622084).** Hypermethylation of the regions 1. (Mirmohammadsadegh et al., 2006)^25^ and 2. (Lahtz et al., 2010)^26^ have been previously associated with transcriptional repression of the *PTEN* gene in melanoma. Hypermethylation of region 3. (Roh et al., 2016, same as region 1.)^27^ was associated with worse survival in melanoma patients.
